# CRISPR’s cutaneous comeback: edited keratinocyte hyperplasias mimicking extramammary Paget’s disease in rare vulvar squamous cell carcinoma

**DOI:** 10.1097/MS9.0000000000004279

**Published:** 2025-11-11

**Authors:** Pakeeza Saif, Muhammad Talha, Mahnoor Fatima, Muammar Hassan, Abdullah Imtiaz

**Affiliations:** a Department of Medicine, King Edward Medical University, Lahore, Pakistan; b Department of Medicine, Shaheed Ziaur Rahman Medical College, Rajshahi Medical University, Rajshahi, Bangladesh; c Department of Medicine, Wah Medical College, Wah Cantt, Pakistan

*To the Editor,*

CRISPR gene editing has made significant advances in treatments and research applications concerned with cutaneous disorders. Evidence from recent research has demonstrated a novel postediting phenomenon: pagetoid spread of keratinocyte hyperplasia mimicking extramammary Paget’s disease (EMPD), exhibited in rare cases of vulvar squamous cell carcinoma, notably without any glandular component. This complex histopathologic mimicry serves as a major diagnostic dilemma in vulvar oncology, creating complications for both clinical management and pathological interpretation^[1,2]^. This is in line with the TITAN Guidelines on the need for transparency in AI use in health care^[3]^.

Currently, the number of research studies is relatively few regarding pseudoinvasive cutaneous changes due to CRISPR, including CRISPR-induced pagetoid hyperplasia. Fewer than 98 publications have currently studied this specific CRISPR-induced dermatologic pseudoinvasion phenomenon, reflecting the evidence gap, with serious implications for precise histopathologic diagnosis and ultimately for the optimization of therapy. However, as gene-editing techniques are becoming an integral part of the dermatology field, it becomes imperative to understand and anticipate these types of pseudoinvasive morphologies^[4]^.

Three-dimensional (3D) bio-printed human skin models provide preclinical platforms specifically designed to examine CRISPR editing results on the keratinocyte behavior and skin architecture directly. These models allow episcopical real-time postedit dynamics to be simulated in detail, aiding the process of differentiating true neoplastic invasion from hyperplastic mimicry caused by gene editing, thereby reducing the misdiagnosis risk considerably. Because of the complex structure of vulvar skin and the peculiar micro-environment, the design and development of vulva-specific modeling and diagnostic parameters has become critically essential^[5]^.

The histopathological pitfalls associated with edited keratinocyte hyperplasias are emphasized by their resemblance to EMPD, a vulvar cancer with distinct glandular features lacking in postedit hyperplasias. Without context-specific knowledge of CRISPR editing effects on epidermal and adnexal cells, the overlap of immune and molecular markers can lead to errors in diagnosis by pathologists. Knowledge of these editing-induced changes is paramount to appropriate surgical and medical management of vulvar squamous neoplasms^[1,2]^.

This perspective illuminates a unique dimension within the discourse of dermatological gene editing, as it documents CRISPR-induced histopathological effects in keratinocytes of the vulvar squamous cell carcinoma area that remains poorly addressed in the current literature. The recognition of postedit pagetoid hyperplasias mimicking EMPD raises important diagnostic issues and highlights the need for interpretive frameworks. These considerations lead to the suggestion of the development of 3D bio-printed full-thickness skin models as an innovative pathway for preclinical validation and an urgent need for vulvar-specific CRISPR editing guidelines to bridge this translational gap. These points provide a wider conversation on safe and precise applications of gene-editing technologies within oncology^[6,7]^.

To conclude, the introduction of CRISPR gene editing highlights the requirement for vulvar-specific guidelines that address keratinocyte hyperplasias mimicking EMPD. Such guidelines should incorporate standard histopatologic criteria, 3D skin models, and interdisciplinary approaches. Interdisciplinary collaboration between oncologists, pathologists, and molecular biologists will effectively reduce diagnostic uncertainty and promote safe and effective application of CRISPR in vulvar dermatologic oncology.

## Data Availability

Not applicable to this study.

## Research registration unique identifying number (UIN)

No human subjects were involved as the type of research being submitted is an editorial.
